# Scaling up implementation of ART: Organizational culture and early mortality of patients initiated on ART in Nairobi, Kenya

**DOI:** 10.1371/journal.pone.0190344

**Published:** 2018-01-02

**Authors:** Richard Ayah

**Affiliations:** School of Public Health, College of Health Sciences, University of Nairobi, KNH, Nairobi, Kenya; University of New South Wales, AUSTRALIA

## Abstract

**Background:**

Scaling up the antiretroviral (ART) program in Kenya has involved a strategy of using clinical guidelines coupled with decentralization of treatment sites. However decentralization pushes clinical responsibility downwards to health facilities run by lower cadre staff. Whether the organizational culture in health facilities affects the outcomes despite the use of clinical guidelines has not been explored. This study aimed to demonstrate the relationship between organizational culture and early mortality and those lost to follow up (LTFU) among patients enrolled for HIV care.

**Methods and materials:**

A stratified sample of 31 health facilities in Nairobi County offering ART services were surveyed. Data of patients enrolled on ART and LTFU for the 12 months ending 30^th^ June 2013 were abstracted. Mortality and LTFU were determined and used to rank health facilities. In the facilities with the lowest and highest mortality and LTFU key informant interviews were conducted using a tool adapted from team climate assessment measurement questionnaire and competing value framework tool to assess organizational culture. The strength of association between early mortality, LTFU and organizational culture was tested.

**Results:**

Half (51.8%) of the 5,808 patients enrolled into care in 31 health facilities over the 12-month study period were started on ART. Of these 48 (1.6% 95% CI 0.8%-2.4%) died within three months of starting treatment, while a further 125 (4.2% 95% CI 2.1%-6.6%) were LTFU giving an attrition rate of 5.7% (95% CI 3.3%-8.6%). Tuberculosis was the most common comorbidity associated with high early mortality and high LTFU. Organizational culture, specifically an adhocratic type was found to be associated with low early mortality and low LTFU of patients enrolled for HIV care (P = 0.034).

**Conclusion:**

The use of ART clinical guidelines in a decentralized health systems are not sufficient to achieve required service delivery outcomes. The attrition rate above would mean 85,000 Kenyans missing care based on current HIV disease burden figures. Deliberate efforts to improve individual health facility leadership and inculcate an adhocratic culture may lower mortality and morbidity associated with initiating ART.

## Introduction

Decentralization of HIV treatment and care in sub-Saharan Africa has been relatively successful despite health systems being under-resourced. Yet to achieve ‘90-90-90’ targets there is a need to expand population coverage and improve service levels, while optimizing resource use [1]. About half of the 36 million people living with HIV are found in East and Southern Africa and two-thirds of the people on highly active antiretroviral therapy (HAART) are found in this region [2]. Despite these high disease burden and health system constraints, the proportion of patients started on HAART is similar at 6–24 months to those from developed countries [3]. However African patients have a high early mortality and patients lost to follow up (LTFU) soon after starting antiretroviral therapy attributable to advanced disease stages at start of treatment [3–6].

Very early mortality is defined as deaths occurring within 3 months of starting ART. Mortality is strongly associated with low baseline CD4 cell count and WHO stage 4 disease with opportunistic infections appearing to be the leading causes of death [4,6]. In addition, the healthcare delivery site is an independent risk factor even after adjustment for these individual patient level factors [7,8]. Factors such as long waiting times for clients; using staff who do not have specialized training in HIV care; failure to contact individuals who do not attend clinic; and lack of communication between pharmacy and clinic staff if ART drugs are not collected lead to patients being lost to follow up [8].

Clinicians are expected to use evidence based guidelines to assess and prepare patients effectively, use ARV drugs rationally, ensure regular and adequate monitoring of patients while recognizing personal and institutional limitations to manage particular ARV and HIV- associated complications and refer or consult appropriately [9]. Following ART initiation, patients are required to be followed up closely for the first 3 months [9,10]. An individual on ART is considered LTFU when they have been absent from the HIV treatment clinic for more than three months [11]. Patients lost to follow up experience higher mortalities than those who adhere to treatment and between 20% to 60% of those lost to follow up have died [12,13].

In 2015, WHO recommended that ART should be initiated in everyone living with HIV at any CD4 cell count [14]. The health system capacity to improve service delivery depends not only on having a health workforce equipped with the necessary skills and equipment but also having an organizational culture that is able to use rewards and incentives to promote the right service delivery values [15]. Scaling up healthcare under such circumstances therefore needs to be innovative and be based on rigorous local strategies that ensure the best possible results are achieved from new investments [15,16].

Kenya is one of four HIV high burden (adult prevalence 6.3%) countries in Africa with Nairobi County, the capital contributes 11.7% of the HIV burden in Kenya [17,18]. When ART availability was limited, programs had stringent entry criteria, requiring good adherence to visits and to preventive therapy before ART was started [19]. But in line with WHO recommendations there has been a dramatic scaling up with ART service delivery points increased from just 15 in 2003 to over 1,829 by 2013 [20,21]. This rapid decentralization of care has pushed clinical responsibility lower down the system requiring strong monitoring and evaluation of clinical performance, that is, clinical governance. However cultural and organizational differences can lead to legitimate variations in recommendations, even if the evidence base is the same [22].

The extent to which there is strong clinical governance therefore depends not only on the availability of clinical guidelines but also on the organizational structure within which the healthcare teams are formed and how the guidelines are used. An ideal organizational structure would ensure clinical mentoring, leadership development and staff appraisal and review regularly clinical processes and outcomes in a systematic way [23].How well clinicians are able to monitor their clinical performance depends on their clinical competence, the organizational culture and the interplay between the two, that is motivation.

**Fig 1:**
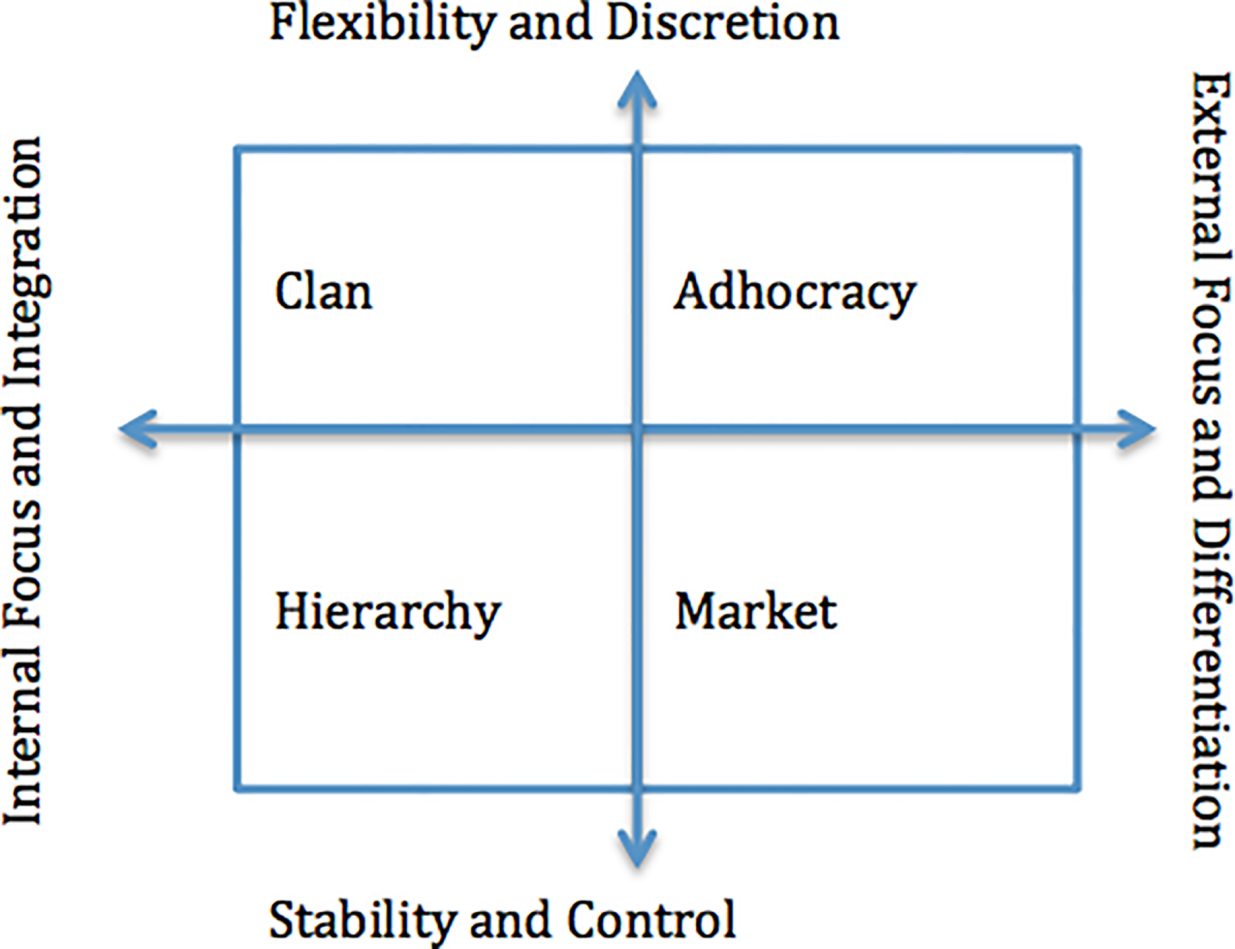
Types of organizational culture.

Organizational culture relates multiple aspects of what is shared among people within the same organisation: for example beliefs, values, norms of behaviour, routines, traditions and sense-making [24].The type of organizational culture determines whether the organization is effective or not in meeting its core mission [25]. Cameron and Quinn in their competing values framework identified four types of organizational culture namely; clan, adhocracy, bureaucracy and market that compete in terms of the values they emphasize, namely cohesion, innovation, stability and competitiveness respectively [26,27] Fig 1.

The typical healthcare organizations will not fall neatly into any one quadrant yet organizational culture has been shown to contribute to variation between health care organizations in performance and outcomes, including innovation, patient satisfaction, health care quality and safety, and employee job satisfaction [28].Measuring group organizational culture using the competing value framework has been shown to distinguish between workplaces and its associations with mental health and well-being outcomes [29]. Early mortality and number of patients LTFU are two important outcome measures of success of an ART program[30]. Three interacting groups of factors affect patient outcomes; patient factors including sex, socioeconomic status, disease staging and co-morbidities; the treatment regimen, when it is started and how the patient responds to it; and how the health services are organized to deliver care [31,32].

Studies of which interventions enhance adherence present a mixed picture and there is particularly in sub-Saharan Africa little information on how organizational culture is associated with health care outcomes [33]. The aim of this study was to determine the health facilities’ cultural factors that contribute to the high early mortality of ART patients. Understanding the culture that underpins the delivery of health services will help managers and policymakers effect positive change in our healthcare institutions to improve on service delivery and therefore reduce ‘early mortality’ and morbidity of ART patients. An understanding of organizational culture in relation to patient outcomes is therefore an important component in the scaling up of HIV treatment and care (HTC) program.

## Methods and materials

### Study setting and participants

A retrospective review of 31 randomly selected health facilities drawn from 141 health facilities in Nairobi County registered to provide outpatient ART services, were stratified into primary (11 health centres and 13 dispensaries) and secondary (7 hospitals) based on expected staffing norms. The study was conducted between 4th August 2013 to 14th October 2013 in two phases [34]. Using a records review tool and patient file abstraction tool, a baseline review of facility registers and patient records for the 12 months ending June 30^th^ 2013 was conducted at these 31 health facilities to determine the early mortality rate and LTFU rate of patients enrolled for the first time. On completion of the baseline, the health facilities were ranked according to their early mortality rate. Then based on the Pareto 80/20 principle that assumes that a small number of causes (20 percent) are responsible for a large percentage (80 percent) of an effect, the bottom quartile (eight) and top quartile (eight) facilities as measured by early mortality and LTFU were then selected for further study to identify the organizational cultural factors associated with the levels of patient mortality and loss to follow [35,36]. Patient files in these 16 facilities of those who had died or LTFU were reviewed and data abstracted to determine presence of any comorbidity (appendix). This was done through key informant interviews using a tool adapted from the competing value framework tool and team climate assessment measurement questionnaire, developed for the National Patient Safety Agency (NPSA) National Health Service UK (appendix) [25,37].

Key informants in each facility were; the health facility head, the person in charge of clinical management of the HIV care program and clinicians providing clinical care. The respondents were asked their perceptions of the behaviour, practices and procedures, both formal and informal that were present within the health facility. A total of 77 interviews were conducted. Exclusion criteria included those clinicians who declined consent and where the interviewee had been employed at the health facility for less than three months. Missing respondents were minimised by making appointments and visiting at least two times where the initial appointment did not take place.

### Data analysis

The primary outcome or endpoint variable was early mortality and LTFU of patients initiated on ART. The main explanatory or predictor variable was organizational culture. Statistical analysis was done using Statistical Products and Service Solutions (SPSS) version 17.0. Univariate analysis was performed to obtain descriptive statistics. Organizational culture was determined using a Likert scale of 1–5; ‘strongly agree, moderately agree, slightly agree/ slightly disagree, moderately disagree and strongly disagree; where 5 was strongly agree and 1 was strongly disagree. Questions that describe the different cultures, that is, adhocracy, bureaucracy, clan and market were grouped together and mean scores and standard deviations calculated for each respondent (appendix). The median (IQR) and mean (SD) were then used to measure variability among the respondents. Pearson’s bivariate correlation was done to examine possible associations between early mortality and LTFU of patients and organizational cultures using Kruskal-Wallis test. Statistical significance was interpreted at 95% confidence level.

Scientific and ethical consent was obtained from the Kenyatta National Hospital/University of Nairobi Ethical and Research Committee (P59/02/2013). For each health facility visited administrative permission was sought before visiting.

## Results

The 31 health facilities when classified by ownership, 18 were government owned,; 10 faith based and non-governmental; and 3 were private (58.1%, 32.3%, 9.7% respectively). All offered standard HIV care and treatment as outlined by national guidelines.

### Patients enrolled in ART

Over the 12-month study period 5,808 patients were enrolled for care. Of those enrolled, 3,009 (51.8%)were started on ART; 48 (1.6% 95% CI 0.8%-2.4%) patients died within three months of starting treatment, while a further 125 (4.2% 95% CI 2.1%-6.6%) patients were lost to follow up. The attrition rate was therefore 5.7% (95% CI 3.3%-8.6%) (Table 1).

**Table 1:** ART enrolment and subsequent attrition rate.

Variable	Overall	Male	Female
**Adults enrolled**			
Mean enrolled per health facility (SD)	187 (178)	63 (61)	124 (119)
Median enrolled per health facility (IQR)	143 (61–236)	42 (20–87)	100 (44–148)
Total enrolled all health facilities	5,808	1,959	3,849
**ART started**			
Mean started per health facility (SD)	94 (20–138)	31 (10–50)	53 (17–90)
Median started per health facility (IQR)	3,009	1,095	1,914
Total started all health facilities	51.9	55.9	49.7
Proportion started on ART	51.9%	55.9%	49.7%
Early Mortality	48 (1.6%)	26 (2.4%)	22 (1.1%)
Loss to Follow Up (LTFU)	125 (4.2%)	50 (4.6%)	75 (3.9%)
Attrition	173 (5.7%)	76 (6.9%)	97 (5.9%)

### Key characteristics of patients who died and those lost to follow up

Descriptive data was available for 40 (83.3%) of the patients who died and 92 (73.6%) of those who were lost to follow up. The average age of those patients who died (35.9 years) or were lost to follow up (35.4) years was the same with no difference when compared by sex. In 45% of patients who died, a co-morbidity was recorded at the start of ART compared to just 18.5% of patients LTFU. Tuberculosis was diagnosed in half (55.6%) of all the patients who died (N = 40) and in 58.8% of those LTFU (N = 92), (Table 2).

**Table 2:** Demographic and co-morbidity characteristics of patients who died and those LTFU.

Variable	Died (n = 40)	Lost to Follow Up (n = 92)
Sex		
Female	21 (52.5%)	51 (55.4%)
Male	19 (47.5%)	41 (44.6)
Median Age	35.9 (SD 16.4)	35.4 (SD 11.6)
Presence of Co-morbidity	18 (45%)	17 (18.5%)
Proportion of patients with comorbidity having TB	10 (55.6%)	10 (58.8%)
Proportion of patients with comorbidity having ‘Other’ comorbidity	8 (44.4%)	7(41.2%)

### HIV care by health facility ownership

Each health facility enrolled a median of 143 adults (IQR 61–236) with private health facilities having the least median number of adults enrolled (51 IQR 48–55), compared to Faith based Organisations (FBO)(85 IQR 35–130) and Government of Kenya (GOK) (196 IQR 75–535) respectively (P = 0.004). The patients enrolled into care did not differ in terms of sex, or levels of co-morbidity at the time of enrolment into care. The number started on ART was found to be significantly different across ownership of the facility with GOK starting a median of 130 patients compared to 40 (IQR13-94) and 28 (IQR 19–43) patients started in FBO and private facilities respectively (P = 0.007). However, there was no significant difference in the proportion of patients started on ART with FBO health facilities starting therapy on 51% of patients enrolled compared to 58% and 54% for private and government health facilities respectively (P = 0.737). There was no significant difference in the mortality rate and lost to follow up rate between the different ownership types.

### HIV care by health facility level

Compared to the overall average of 5.7%, hospitals had an attrition rate of 5.6%(95% CI 0.5%-16.3%), dispensaries 6.4%%(95% CI 2.3%-10.5%), and Health Centres the lowest at 5.3% %(95% CI 2.6%-9.4%) but there was no significant difference in the either mortality or LTFU when categorized by facility type even though lower proportions of patients enrolled at dispensaries were started on ART, (P = 0.052).

### Health facility clinical governance structure

On completion of the baseline, 77 interviews were held with key informants and clinicians from the top and bottom quartiles of the health facilities as measured by early mortality and LTFU. Two thirds of the respondents were female (53) and one third male (24). In terms of age (n = 53), the median age of respondents was 36 years with the youngest being 24 years and the oldest 63 years. The largest group of respondents were nurses (41%), followed by clinical officers (34%) and administrators (16%). Clinical managers without cadre specification were few (4%) as were doctors (1%). Almost all (92%) had supervisory responsibility with 20% managing 1–3 persons, 14% managing 4–6, just 7% managing 7–9 persons and 27% having more than nine persons to supervise (n = 56). Just over half (55%) had received one job promotion in the last five years, while a third (31.2%) had received two promotions, with 7% having been promoted three times and a lucky 5% having more than moved up more than four times. There were no significant differences or associations between individual characteristics such as cadre, age, sex, work experience of staff and patient outcomes.

**Fig 2:**
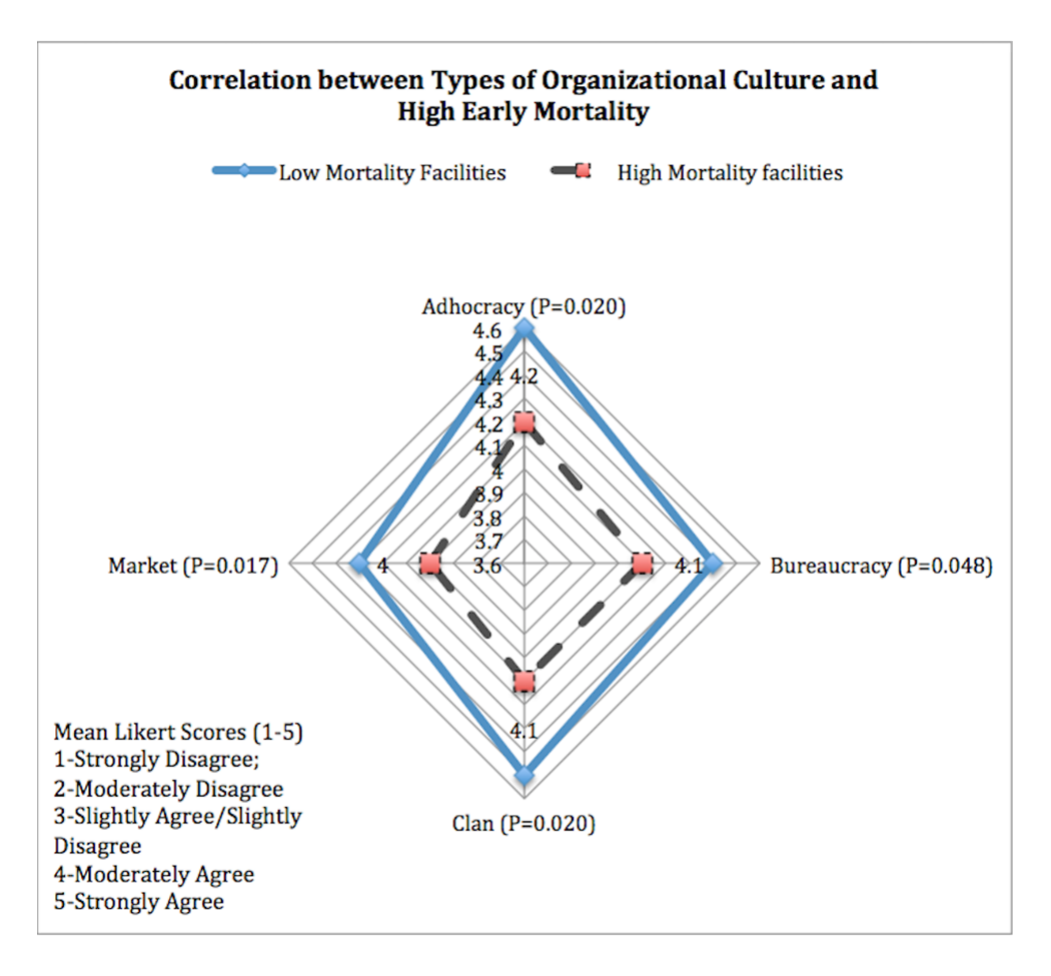
Correlating types of organizational culture and early mortality.

Health facilities reporting low early mortality had significant differences in organizational culture with those health facilities with higher early mortality (Fig 2).

Task reflexivity, the degree to which health facility staff review their objectives, their ways of achieving them and their methods of working was scored the highest across all health facilities, while team stability was found to be low. Hospitals (3.8 SD 1.2) were reported to have higher levels of team stability (P = 0.015) compared to health centres (2.0 SD 1.2) and dispensaries (2.0 SD 0.9). Managers in health centres (4.5 SD 0.3) and dispensaries (4.4 SD 0.3) rated themselves more highly (P = 0.015) than those at hospitals, (3.8 SD 0.5). When the organizational culture was mapped against early high mortality, the extent of inter-professional learning emerged as a significant factor with lower rates of learning associated with higher early mortality rates (P = 0.006 95% CI -2.6 - -0.1,Pearson’s correlation -0.567). Overall having a strong organizational culture was associated with low mortality rates (P = 0.020 Pearson’s correlation -0.432).

An adhocracy-type culture was reported at much higher levels in facilities with low LTFU compared to those with high LTFU (P = 0.034 4.6 SD 0.2 and 4.3 SD0.2 respectively) (Pearson’s correlation -0.639). Health facilities with an adhocratic culture in addition to having an all round strong organizational culture were associated with a low early mortality rate (p = 0.020 4.6 SD 0.2 and 4.2 SD0.4). The adhocratic culture association was independent of the facility ownership or type of health facility.

## Discussion

The aim of this study was to identify the health organizational cultural factors that were associated with low early mortality of ART patients. An adhocracy type of culture, which is associated with adaptation and innovation, was found to be associated with low early mortality and low levels of lost to follow up of patients. Having a culture open to innovation has been demonstrated for example in ‘adherence clubs’ set up in South Africa for stable ART patients [16]. However patient outcomes in these adherence clubs have yet to be evaluated and this study potentially points to what kind of results may be achieved.

Late patient presentation, low CD4 count, being male and low socio-economic status have been cited as factors contributing to early deaths and high attrition rates of patients initiated on HAART [32,38,39]. Revised WHO treatment guidelines addressed the issue of early treatment with recommendations of earlier ART initiation threshold [40]. This study’s overall attrition rate of 5.7% is comparable to the 8% found elsewhere in Kenya [11]. Other studies suggest that adherent patients are less likely to suffer virological failure, a sign of treatment failure [41]. The early mortality rate was low at 1.6%, compared to rates of 3.3% a month reported by Rosen et al in a systematic review of Sub-Saharan Africa ART programs and 8.9% in rural Malawi [6,31].

In this study, there was no difference in the type of patient enrolled into HIV care when compared by health facility level or the presence of co-morbidity at the point of enrolment. Nor was there any difference in patients started on ART when comparing by health facility level or ownership. The similarity in patient outcomes may be explained by reported high levels of external supervision and training at dispensaries (94%, 83%) and health centres (92% and 95%) despite the lower cadre levels at the primary healthcare level [42],[43].

Among patients who died, 45% had a co-morbidity with tuberculosis (55.6%) the leading comorbidity. TB is a major cause of HIV-associated morbidity and mortality [9,10]. HIV patients diagnosed with TB are according to guidelines meant to trigger intensified case finding and therefore the attrition rate should be low. Among patients lost to follow up with a comorbidity, 58.8% had TB reaffirming that having TB is associated with early deaths and loss to follow up and that clinical staff need to improve on TB management [44–46].

Patient and disease factors alone do not explain well why some organizations are more successful than others that provide the same service. The health facility organizational social context directly affects service quality and outcomes [47]. The scaling up of ART has changed the management of the condition with patients in need of life-long regular follow-up. The required organizational culture would therefore be one able to adapt to the increase in workload in maintaining large numbers of patients on ART. Health facilities with high rates of LTFU had weaker organizational culture compared to those where the LTFU was low, significantly in relation to bureaucracy, market and adhocratic types of culture, less so with regard to clan culture. Those health facilities that placed less emphasis on teamwork, participation, and consensus (Clan); did not encourage individual initiative and freedom (adhocracy); had less focus on competitive actions and achievement of measurable goals (market); and had less formal rules and policies to hold the organization to ensure long term efficient smooth operations (bureaucracy).

The study results show that clan culture is important but not enough to sustain the desired level of quality of care. Clan culture offers a structure to account for the complex social systems and social norms found in healthcare, where professional autonomy and peer control is central to how work is done [48]. Bureaucracies work well where a top-down, planned program to deliver a uniform public service is required. Public sector successes include immunization and literacy campaigns [49].The use of standard treatment protocols in the rapid scale up of ART requires at least a bureaucratic organizational structure to succeed. However in the typical healthcare provision setting a command and control structure alone as suggested by a bureaucracy is unlikely to be adequate because of the dynamic nature of providing healthcare [50].

The managing authority of the health facility is often taken to be an important factor in health service delivery with service assessments using ownership as a key service delivery differentiator [43,50]. However facility size maybe more important than ownership in determining service delivery outcomes with small-scale health providers, mainly for profit providers, struggling to remain financially viable [50]. This study demonstrates that in terms of patient characteristics and outcomes there is no difference between faith-based, public and private for profit health facilities. This finding is similar to a systematic review of hospital ownership and quality of care in the United States that found that there was as much heterogeneity among hospitals of the same ownership form as across ownership forms and that individual study result differences were often a result in differences in study analytical methods rather than ownership differences [51]. The implication of these results is that policymakers need to pay more attention to individual health facilities outputs and outcomes rather than grouping them by ownership to determine expected quality of care.

### Study limitations

Random sampling reduced the possibility of non-coverage bias despite health centres and to a lesser extent hospitals being relatively over-sampled; however the sample number of dispensaries obtained was close to the national average of 49% of all health facilities. Eighteen facilities declined to participate, but they were managed by just two owners one private, the other faith based and it is possible that the response rate of 55% has lead to an underestimation of the magnitude of mortality and LTFU. However given that the facilities represented just two owners it is possible that the non-response may not be an under representation of the population of study [52]. Nationally NGO managed health facilities are just 3% of the total number of health facilities [43]. The key informants chosen were the immediate supervisors rather than all staff because there is evidence that there may be problems applying the conventional competing value framework subscales to non-supervisory staff [53]. Patients lost to follow up may die or re-enter the health system at a later date and so leading to an under or overestimate of the LTFU. Limitations to this analysis include the potential omission or random misclassification due to clinician error in recording of patient diagnosis affecting the mortality and morbidity analysis.

## Conclusion

This study demonstrates that the type of health facility’s organizational culture specifically a weak adhocratic culture is associated with high early mortality rates. In a decentralized system with rapid scaling up of services the use of clinical guidelines alone coupled with individual training of clinicians is not sufficient to maintain required health outcomes. Deliberate efforts to empower individual health facility leaders, to inculcate an adhocratic culture embracing a culture of innovation, sharing of knowledge and being accountable should improve service delivery outcome. Further policy makers need to classify and monitor health facilities by level of service and size not just ownership.

## Supporting information

S1 Appendix Org culture data abstraction tool1. (DOCX)

S2 Appendix Org culture data abstraction tool2. (DOCX)

S3 Appendix Org culture data abstraction tool3. (DOCX)

S4 Appendix Org culture data set 1. (SAV)

S5 Appendix Org culture mortality data. (SAV)

S6 Appendix NRLS-1058 TC template teamworking. (XLS)

S7 Appendix NRLS 1058 TCAM 2006 v1. (PDF)

S8 Appendix Scoring analysis org culture. (XLSX)
