# Supplementary material for: Scaling up implementation of ART: Organizational culture and early mortality of patients initiated on ART in Nairobi, Kenya
Source: PLoS One. 2018 Jan 2;13(1):e0190344. doi: 10.1371/journal.pone.0190344 (PMC5749788; doi:10.1371/journal.pone.0190344)
Supplement: S1 Appendix — (DOCX) [file pone.0190344.s001.docx]

|  | 1. Patients enrolled in HIV care last 15 months | | | 1. Total patients started on ART last 15 months | | |
| --- | --- | --- | --- | --- | --- | --- |
|  | Total | Male | Female | Total | Male | Female |
| April 2012 |  |  |  |  |  |  |
| May 2012 |  |  |  |  |  |  |
| June 2012 |  |  |  |  |  |  |
| July 2012 |  |  |  |  |  |  |
| Aug 2012 |  |  |  |  |  |  |
| Sep 2012 |  |  |  |  |  |  |
| Oct 2012 |  |  |  |  |  |  |
| Nov 2012 |  |  |  |  |  |  |
| Dec 2012 |  |  |  |  |  |  |
| Jan 2013 |  |  |  |  |  |  |
| Feb 2013 |  |  |  |  |  |  |
| Mar 2013 |  |  |  |  |  |  |
| Apr 2013 |  |  |  |  |  |  |
| May 2013 |  |  |  |  |  |  |
| Jun 2013 |  |  |  |  |  |  |

Is the patient register for patients enrolled into HIV complete up to yesterday? Yes / No

|  | 1. Patients enrolled in ART who died within 3 months of enrolment | | | 1. Patients enrolled in ART who are lost to follow up within 3 months of enrolment | | | Transfers  Out |
| --- | --- | --- | --- | --- | --- | --- | --- |
|  | Total | Male | Female | Total | Male | Female |  |
| April 2012 |  |  |  |  |  |  |  |
| May 2012 |  |  |  |  |  |  |  |
| June 2012 |  |  |  |  |  |  |  |
| July 2012 |  |  |  |  |  |  |  |
| Aug 2012 |  |  |  |  |  |  |  |
| Sep 2012 |  |  |  |  |  |  |  |
| Oct 2012 |  |  |  |  |  |  |  |
| Nov 2012 |  |  |  |  |  |  |  |
| Dec 2012 |  |  |  |  |  |  |  |
| Jan 2013 |  |  |  |  |  |  |  |
| Feb 2013 |  |  |  |  |  |  |  |
| Mar 2013 |  |  |  |  |  |  |  |
| Apr 2013 |  |  |  |  |  |  |  |
| May 2013 |  |  |  |  |  |  |  |
| Jun 2013 |  |  |  |  |  |  |  |

Comments

1. Does the health facility have a PMTCT program Yes /No

*If yes then fill in tables 3 and 4 below. If no then proceed*

| **PMTCT** | 1. Infants enrolled in HIV care last 15 months | | | 1. Total infants started on ART last 15 months | | |
| --- | --- | --- | --- | --- | --- | --- |
|  | Total | Male | Female | Total | Male | Female |
| April 2012 |  |  |  |  |  |  |
| May 2012 |  |  |  |  |  |  |
| June 2012 |  |  |  |  |  |  |
| July 2012 |  |  |  |  |  |  |
| Aug 2012 |  |  |  |  |  |  |
| Sep 2012 |  |  |  |  |  |  |
| Oct 2012 |  |  |  |  |  |  |
| Nov 2012 |  |  |  |  |  |  |
| Dec 2012 |  |  |  |  |  |  |
| Jan 2013 |  |  |  |  |  |  |
| Feb 2013 |  |  |  |  |  |  |
| Mar 2013 |  |  |  |  |  |  |
| Apr 2013 |  |  |  |  |  |  |
| May 2013 |  |  |  |  |  |  |
| Jun 2013 |  |  |  |  |  |  |

| **PMTCT** | 1. Patients enrolled in ART who died within 3 months of enrolment | | | 1. Infants enrolled in ART who are lost to follow up within 3 months of enrolment | | | Transfers  Out |
| --- | --- | --- | --- | --- | --- | --- | --- |
|  | Total | Male | Female | Total | Male | Female |  |
| April 2012 |  |  |  |  |  |  |  |
| May 2012 |  |  |  |  |  |  |  |
| June 2012 |  |  |  |  |  |  |  |
| July 2012 |  |  |  |  |  |  |  |
| Aug 2012 |  |  |  |  |  |  |  |
| Sep 2012 |  |  |  |  |  |  |  |
| Oct 2012 |  |  |  |  |  |  |  |
| Nov 2012 |  |  |  |  |  |  |  |
| Dec 2012 |  |  |  |  |  |  |  |
| Jan 2013 |  |  |  |  |  |  |  |
| Feb 2013 |  |  |  |  |  |  |  |
| Mar 2013 |  |  |  |  |  |  |  |
| Apr 2013 |  |  |  |  |  |  |  |
| May 2013 |  |  |  |  |  |  |  |
| Jun 2013 |  |  |  |  |  |  |  |
