# Supplementary material for: Scaling up implementation of ART: Organizational culture and early mortality of patients initiated on ART in Nairobi, Kenya
Source: PLoS One. 2018 Jan 2;13(1):e0190344. doi: 10.1371/journal.pone.0190344 (PMC5749788; doi:10.1371/journal.pone.0190344)
Supplement: S2 Appendix — (DOCX) [file pone.0190344.s002.docx]

1. **For each patient on ART who has been identified as having died within 3 months of starting ART, fill in the form below.**

| Patients enrolled in ART who died within 3 months of enrolment | | | |
| --- | --- | --- | --- |
| Patient Number |  |  |  |
| Sex | 1. Female | 2. Male |  |
| Age |  | Date of last birthday |  |
| Date of enrollment for HIV/Care |  |  |  |
| Date of Start of ART |  |  |  |
| Co-morbidity | 1. Yes | 2. No |  |
| Type of Co-morbidity | 1. TB | 2. PCP | 3. Other |
| Clinician (Code) attending on date of enrolment |  |  |  |
| Treatment Regimen | Regimen 1 | Regimen 2 | Regimen 3 |

**For each patient on ART who has been identified as having been lost to follow up within 3 months of enrolment, fill in the form below.**

| Patients enrolled in ART who are lost to follow up within 3 months of enrolment | | | |
| --- | --- | --- | --- |
| Patient Number |  |  |  |
| Sex | 1. Female | 2. Male |  |
| Age |  | Date of last birthday |  |
| Date of enrollment for HIV/Care |  |  |  |
| Date of Start of ART |  |  |  |
| Co-morbidity | 1. Yes | 2. No |  |
| Type of Co-morbidity | 1. TB | 2. PCP | 3. Other |
| Clinician (Code) attending on date of enrolment |  |  |  |
| Treatment Regimen | Regimen 1 | Regimen 2 | Regimen 3 |
