# Supplementary material for: Scaling up implementation of ART: Organizational culture and early mortality of patients initiated on ART in Nairobi, Kenya
Source: PLoS One. 2018 Jan 2;13(1):e0190344. doi: 10.1371/journal.pone.0190344 (PMC5749788; doi:10.1371/journal.pone.0190344)
Supplement: S3 Appendix — (DOCX) [file pone.0190344.s003.docx]

**HEALTH FACILITY CLINICAL GOVERNANCE CHECKLIST**

| District____________ Facility Code_____ Date____/___/___  Facility Name________________ Address________________________________________________________________________  Facility Ownership/Affiliation 1. MoH 2. FBHS 3. Private (specify)  Tel__________________________________ Email__________________________________  Health facility level 1-6 |
| --- |
| Name of Interviewer_______________________  Name of the Respondent:___________________________________Profession_____________  Position______________ ______Tel ____________ Email__________________  Date appointed to current position_____________________ Time in current position (in months)___________________ |

*Kindly describe your role and position .I am interested in understanding this health facility is organized. Specifically I would like to know how the clinical team is organized.*

| **Clinical Meetings** | **Yes** | **No** |
| --- | --- | --- |
| 1. Are clinical meetings held at this facility? |  |  |

*(A clinical meeting is where patient outcomes and outputs are discussed)*

1. Who is required to attend such meetings? (Mark one or more than one)

| Nurses |  |
| --- | --- |
| Doctors |  |
| Clinical Officers |  |
| Lab technicians |  |
| Administrators |  |
| Other (specify |  |

|  | Yes | No |
| --- | --- | --- |
| 1. Is attendance by all clinical staff mandatory? |  |  |

|  | Daily | Weekly | Monthly | Quarterly | Ad-hoc |
| --- | --- | --- | --- | --- | --- |
| 1. How often are these meetings held? |  |  |  |  |  |

| 1. On what date was the last meeting held? |  |
| --- | --- |

*Confirm above questions by looking at the minutes of meetings. If the date on the minutes of the last meeting differ from the verbal answer given above then indicate the date of last meeting by minutes. ______________________________*

|  | Yes | No | I don’t know |
| --- | --- | --- | --- |
| 1. Are there minutes of the last meeting |  |  |  |

|  | We usually see them in the next meeting | Minutes are sent (email or hardcopy) before next meeting | Minutes are taken but not circulated to members |
| --- | --- | --- | --- |
| 1. If Yes to question 6 then when are minutes availed? |  |  |  |

**Teamwork**

Please mark the box most applicable to your opinion

| 1 | Strongly Disagree |
| --- | --- |
| 2 | Moderately Disagree |
| 3 | Slightly Agree/Slightly Disagree |
| 4 | Moderately Agree |
| 5 | Strongly Agree |

| **Task Reflexivity** | **1** | **2** | **3** | **4** | **5** |
| --- | --- | --- | --- | --- | --- |
| 1. The team often reviews its objectives |  |  |  |  |  |
| 1. We regularly discuss whether the team is working effectively together |  |  |  |  |  |
| 1. How well we communicate information is often discussed |  |  |  |  |  |
| 1. The team often reviews its approach to getting the job done |  |  |  |  |  |
| 1. The way decisions are made in this team is often reviewed |  |  |  |  |  |
| 1. We regularly discus whether we effectively manage issues of patient safety together |  |  |  |  |  |
| 1. The team helps me to question my own beliefs and behaviours relevant to patient safety. |  |  |  |  |  |

| **Participative Trust and Safety** | **1** | **2** | **3** | **4** | **5** |
| --- | --- | --- | --- | --- | --- |
| 1. Members of this team are able to bring up problems and tough issues |  |  |  |  |  |
| 1. People in this team sometimes reject others for being different |  |  |  |  |  |
| 1. It is difficult to ask other members of this team for help |  |  |  |  |  |
| 1. No one in this team would deliberately act in a way that undermines my efforts |  |  |  |  |  |
| 1. Working with members of this team my unique skills and talents are valued and utilized |  |  |  |  |  |
| 1. In this team it is difficult to speak up if I perceive a problem with patient care |  |  |  |  |  |

| **Team Learning behaviour** | **1** | **2** | **3** | **4** | **5** |
| --- | --- | --- | --- | --- | --- |
| 1. We regularly take time to consider ways of improving our team’s work processes |  |  |  |  |  |
| 1. The team tends to handle differences of opinion privately rather than addressing them directly as a group |  |  |  |  |  |
| 1. This team frequently seeks new information that leads us to make important changes |  |  |  |  |  |
| 1. In this team someone always makes sure that we stop to reflect on the team’s work process |  |  |  |  |  |
| 1. People in this team often speak up to test assumptions about issues under discussion |  |  |  |  |  |
| 1. We invite people from outside the team to present information or have discussions with us |  |  |  |  |  |
| 1. We invite managers to present information or have discussions with us |  |  |  |  |  |

| **Individual development** | **1** | **2** | **3** | **4** | **5** |
| --- | --- | --- | --- | --- | --- |
| 1. The team supports my professional development |  |  |  |  |  |
| 1. The team supports my individual learning activities |  |  |  |  |  |
| 1. The team provides me with useful ideas and practical support |  |  |  |  |  |
| 1. We pay attention to each others work so that the work done remains at a high standard |  |  |  |  |  |
| 1. We pay attention to each other’s behaviour relevant to patient safety |  |  |  |  |  |

| **Inter-professional Credibility** | **1** | **2** | **3** | **4** | **5** |
| --- | --- | --- | --- | --- | --- |
| 1. I am comfortable accepting procedural suggestions from other team members |  |  |  |  |  |
| 1. I trust that other members’ knowledge about the program is credible |  |  |  |  |  |
| 1. I am confident about relying on the information that other team members bring to the discussion |  |  |  |  |  |
| 1. When other team members give information, I want t double check it for myself |  |  |  |  |  |
| 1. I do not have faith in the other team members’ “expertise” |  |  |  |  |  |
| 1. Input from junior members of the team about patient care is listed to in the team |  |  |  |  |  |
| 1. I am comfortable accepting suggestions about patient care from other team members |  |  |  |  |  |

| **Inter-professional learning** | **1** | **2** | **3** | **4** | **5** |
| --- | --- | --- | --- | --- | --- |
| 1. There is often conflict between professional groups about how best to achieve our objectives |  |  |  |  |  |
| 1. There is a feeling of trust and safety between colleagues with different professional backgrounds |  |  |  |  |  |
| 1. There is a climate of constructive debate between professional groups within the group |  |  |  |  |  |
| 1. There is often conflict between professional groups about how best to achieve quality patient care. |  |  |  |  |  |
| 1. All professional groups work closely together to ensure quality patient care |  |  |  |  |  |

| **Regular contact** | **1** | **2** | **3** | **4** | **5** |
| --- | --- | --- | --- | --- | --- |
| 1. There is regular contact among group members |  |  |  |  |  |
| 1. We hold group meetings regularly |  |  |  |  |  |
| 1. We are regularly in touch with each other |  |  |  |  |  |
| 1. We have frequent and mutual exchanges |  |  |  |  |  |
| 1. The group members meet frequently to discuss topics informally |  |  |  |  |  |

| **Mutual Trust** | **1** | **2** | **3** | **4** | **5** |
| --- | --- | --- | --- | --- | --- |
| 1. There is trust and friendliness among group members |  |  |  |  |  |
| 1. We support each other |  |  |  |  |  |
| 1. Among group members there is a sense of helpfulness |  |  |  |  |  |
| 1. Among group members there is understanding and empathy |  |  |  |  |  |
| 1. There is a lack of team work in this work group |  |  |  |  |  |

| **Team Efficacy** | **1** | **2** | **3** | **4** | **5** |
| --- | --- | --- | --- | --- | --- |
| 1. Certain individuals in this team lack the special skills needed for good team work |  |  |  |  |  |
| 1. Members of this team have more that enough talent and experience to ensure quality patient care |  |  |  |  |  |
| 1. Everyone in this team has the special skills needed to ensure quality patient care |  |  |  |  |  |

| **Team stability** | **1** | **2** | **3** | **4** | **5** |
| --- | --- | --- | --- | --- | --- |
| 1. There is a high turnover of staff in this team   *(changes more than 1 person within last 6 months)* |  |  |  |  |  |
| 1. I have the impression that many people have left the team over the last 6 months |  |  |  |  |  |

| 1. How many teams do you work in? | 1 | 2 | 3 | 4 | >5 |
| --- | --- | --- | --- | --- | --- |

| **Shared leadership** | **1** | **2** | **3** | **4** | **5** |
| --- | --- | --- | --- | --- | --- |
| 1. A number of people lead this team |  |  |  |  |  |
| 1. We all have leadership roles in one way or another |  |  |  |  |  |
| 1. There is a very clear leader |  |  |  |  |  |
| 1. There is no clear leader |  |  |  |  |  |
| 1. There is conflict over who leads the team |  |  |  |  |  |
| 1. Team members take initiative to promote high shared motivation and commitment |  |  |  |  |  |
| 1. Team members take initiatives to help the work group build and use members’ knowledge and skills |  |  |  |  |  |
| 1. Team members take initiatives to constructively resolve problems or conflicts that develop among members |  |  |  |  |  |
| 1. Team members tell other members what to do and how they should do it. |  |  |  |  |  |

If you work in more that one team, please answer the questions below in relation to the team you have been considering through this questionnaire.

1. How would you categorize the type of team that you have answered the above questions about?

Multi-disciplinary

Uni-disciplinary

|  | 2-5 | 6-9 | 10-15 | >15 |
| --- | --- | --- | --- | --- |
| 1. How many people work in this team? |  |  |  |  |

|  | **Yes** | **No** |
| --- | --- | --- |
| 1. Have you attended any professional training (earning CPD points) in the last one year? *(july 2012 – Jun 2013)* |  |  |

|  | 1 | 2 | 3 | 4 | 5 | >5 |
| --- | --- | --- | --- | --- | --- | --- |
| 1. If yes, how many trainings? |  |  |  |  |  |  |

|  | **Yes** | **No** |
| --- | --- | --- |
| 1. Are there standard operating procedures for management of patients initiating ART? |  |  |

| 1. If yes where are the SOP derived from? |  |
| --- | --- |
| National ART guidelines 2011 | 1 |
| National ART guidelines 2011 | 2 |
| Health Facility has own written guidelines | 3 |
| Individual Clinicians treat patients using their own clinical judgement | 4 |
| Other (specify) | 5 |
