# Supplementary material for: Scaling up implementation of ART: Organizational culture and early mortality of patients initiated on ART in Nairobi, Kenya
Source: PLoS One. 2018 Jan 2;13(1):e0190344. doi: 10.1371/journal.pone.0190344 (PMC5749788; doi:10.1371/journal.pone.0190344)
Supplement: S7 Appendix — (PDF) [file pone.0190344.s007.pdf]

aston organisation development

NHS
National Patient Safety Agency
National Reporting and Learning Service

# Template for the TCAM Report

This template is to be used when generating the initial and final TCAM Reports.

## Process for generating a TCAM Report

- ❑ Collect the completed questionnaires from all team members.
- ❑ Download two files from the NPSA website:
  - the '*Template for Scoring TCAM*' Excel file
  - the '*Template for the TCAM Report'* Word file.
- ❑ Enter the response scores for each team member into the '*Template for Scoring TCAM*' Excel file. The spreadsheet will automatically calculate the scores for the 11 Dimensions, and the Patient Safety components.
- ❑ Enter the 'Team' scores from the '*Template for Scoring TCAM*' Excel file into the relevant sections in the '*Template for the TCAM Report'* Word file to produce the TCAM report.
  - If you are producing the final TCAM Report, enter the initial scores in italics and the final scores in bold to enable team members to see the difference.

© Aston Organisation Development Ltd 2006 & NPSA 2006

# The TCAM Report

| Team Organisation | | | | | | |
|---|---|---|---|---|---|---|
| ***Lower Scores*** | ***1*** | ***2*** | ***3*** | ***4*** | ***5*** | ***Higher Scores*** |
| 1 Team Stability | | | | | | |
| There is high turnover of staff in the team. | | | | | | There is low turnover of staff in the team. |
| 2 Effective Leadership | | | | | | |
| Leadership of the team is not clear and Team Members do not share leadership. There is little peer coaching within the team. | | | | | | Leadership of the team is clear and shared. There is a good deal of peer coaching within the team. |
| 3 Regular Contact | | | | | | |
| There is very little regular formal or informal contact between Team Members. | | | | | | Team Members meet regularly and frequently, both formally and informally. |
| Patient Safety Component | | | | | | |
| There is very little discussion about patient safety issues. Meetings about patient safety or adverse incident management rarely take place. | | | | | | Team Members frequently discuss patient safety issues and adverse incident management, both formally and informally. |
| 4 Team Efficacy | | | | | | |
| The team goal is either not achievable with the skills available, or unreasonable time or effort is required to achieve the team's goal. | | | | | | The team has the skills to achieve its goals with ease. |
| Patient Safety Component | | | | | | |
| The team cannot achieve high standards of patient safety with the current skills, time or resources available to it. | | | | | | The team has the skills, time and resources available to achieve high standards of patient care with ease. |
| 5 Task Reflexivity | | | | | | |
| The team rarely reviews team objectives, processes or effectiveness. | | | | | | The team regularly takes time out to formally review team objectives, processes and effectiveness. |
| Patient Safety Component | | | | | | |
| The team rarely reflects upon adverse incident management or patient safety issues. | | | | | | The team regularly reviews adverse incident management and patient safety issues. |

© Aston Organisation Development Ltd 2006 & NPSA 2006

## Team Culture

| Lower Scores | 1 | 2 | 3 | 4 | 5 | Higher Scores |
|---|---|---|---|---|---|---|
| **6 Participative Trust and Safety** | | | | | | |
| Team Members do not value the skills and talents of others, especially when they are different. Mistakes tend not to be forgiven and people do not often ask for help. | | | | | | Team Members value and rely upon the skills and talents of others, even when they are different. Mistakes are not held against individuals and people often ask each other for help. |
| Patient Safety Component | | | | | | |
| Team Members find it difficult to discuss concerns about patient safety. Reporting of such concerns or problems is not encouraged. | | | | | | Team Members find it easy to discuss concerns about patient safety. Reporting of such concerns and problems is actively encouraged. |
| **7 Mutual Trust** | | | | | | |
| There is little trust, friendliness, support, co-operation, helpfulness or empathy between Team Members. | | | | | | There is a good deal of trust, friendliness, mutual support, helpfulness, co-operation and empathy between Team Members. |
| Patient Safety Component | | | | | | |
| Team Members do not feel confident that they will receive support and co-operation from colleagues in relation to patient safety issues and adverse incident management. | | | | | | Team Members are confident that they will be supported by team colleagues in matters related to patient safety and adverse incident management. |
| **8 Inter-professional Credibility** | | | | | | |
| Team Members have low or inaccurate perceptions of the skills and expertise of others. | | | | | | Team Members value and rely on the skills and expertise of others and their assessments are accurate. |
| Patient Safety Component | | | | | | |
| Team Members have little faith in the ability of others to ensure patient safety. Junior members of the team are not listened to when they express concern about patient safety issues. | | | | | | Team Members have faith in the ability of others to ensure patient safety. The views of junior members of the team in relation to patient care and safety issues are valued. |

© Aston Organisation Development Ltd 2006 & NPSA 2006

| 9 Individual Development | | | | | | |
|---|---|---|---|---|---|---|
| There is little practical support given to enable individual development. Team Members do not feel that their creativity or learning activities are supported or valued. | | | | | | Team Members' creativity and learning activities are actively supported and they are provided with useful ideas and practical support to optimise learning opportunities. |
| Patient Safety Component | | | | | | |
| Team Members do not pay attention to the behaviour of others that could affect patient safety. | | | | | | Team Members pay attention to each other's patient safety-related behaviour and provide support to enable improvement. |
| 10 Team Learning Behaviour | | | | | | |
| Team Members are discouraged from reviewing work processes and seldom make comments or seek information to inform important changes. There is little sharing of information between Team Members. | | | | | | Team Members regularly review work processes and frequently seek information from others outside the team to inform change and improve team performance. Team Members share valuable information. |
| Patient Safety Component | | | | | | |
| Information from adverse incident reports is very rarely used to bring about improvements in patient safety. | | | | | | Information from adverse incident reports is always used to bring about improvements in patient safety. |
| 11 Inter-professional Learning | | | | | | |
| There is conflict, distrust and little collaboration and individual learning across professional groups. Team Members rarely show interest in the work of those in different professional groups. | | | | | | There is constructive debate, exchange and collaboration across professional groups. Each profession pays attention to the work of others and there is a good deal of support for individual learning between professional groups. |
| Patient Safety Component | | | | | | |
| There is little constructive debate about patient safety issues between professional groups and there is often conflict about how best to ensure patient safety. | | | | | | There is a good deal of constructive debate between professional groups, leading to improved levels of patient care and safety. |

© Aston Organisation Development Ltd 2006 & NPSA 2006
